# Point-of-care Head and Neck Sonography for Clinical Problem-solving: Impact of One-day Training Sessions on Medical Student Education

**DOI:** 10.7759/cureus.3740

**Published:** 2018-12-17

**Authors:** Lucas Friedman, Elaine Situ-LaCasse, Josie Acuna, Richard Amini, Steven C Irving, Lori A Stolz, Robert Sterling, Chan Jung, Arthur B Sanders, Srikar Adhikari

**Affiliations:** 1 Emergency Medicine, University of California, Riverside, USA; 2 Emergency Medicine, University of Arizona, Tucson, USA; 3 Emergency Medicine, University of Cincinnati, Cincinnati, USA; 4 Anesthesiology, University of California, Irvine, USA

**Keywords:** ultrasound, medical students, clinical problem solving, head and neck, sonography

## Abstract

Introduction

The curriculum for medical student education is continuously evolving to emphasize knowledge acquisition with critical problem-solving skills. Medical schools have started to implement curricula to teach point-of-care ultrasound skills. To our knowledge, the expansion into head and neck sonography for medical student education is novel and has never been studied. Our objective was to determine the feasibility of implementing point-of-care head and neck sonography and critical problem-solving instruction for medical student education.

Methods

This was a cross-sectional study enrolling third-year medical students with minimal prior ultrasound experience. A one-day educational curriculum focusing on the use of head and neck ultrasound for clinical problem-solving was integrated into one of the week-long intersessions. The components of point-of-care ultrasound workshop included asynchronous learning, one-hour didactic lecture, followed by a pre-test assessment, then a one-day hands-on workshop, and finally a post-test assessment administered at the end of the training session.

Results

A total of 123 subjects participated in this study. Ninety-one percent completed the questionnaire prior to the workshop and 83% completed the post-test questionnaire. The level of comfort with using an ultrasound system significantly increased from 31% to 92%. Additionally, the comfort level in interpreting ultrasound images also significantly increased from 21% to 84%. Eighty-nine percent (95% CI, 86%-97%) had an interest in learning ultrasound and would enroll in an optional ultrasound curriculum if given the opportunity. Knowledge of specific ultrasound applications also increased from 60% (after asynchronous learning and lectures) to 95% (after additional hands-on sonographic training).

Conclusion

At our institution, we successfully integrated point-of-care head and neck sonography and critical problem-solving instruction for medical student education.

## Introduction

As point-of-care ultrasound becomes a more ubiquitous component of clinical practice across various specialties, it is now being widely introduced into the undergraduate medical school curriculum. A number of medical schools have implemented curricula to teach point-of-care ultrasound skills [1]. However, the methodology for teaching point-of-care ultrasound to medical students remains highly variable among medical schools, without standardized techniques or syllabi. While there are few schools with a well-established four-year curriculum in place, the majority of schools lie elsewhere on the spectrum [2].

The traditional focus of medical schools is to use ultrasound to teach anatomy, physiology, and physical examination skills. However, the curriculum in medical schools has been continuously evolving in an effort to emphasize knowledge acquisition with critical problem-solving skills. A study by Schmidmaier et al. has demonstrated that conceptual knowledge alone is insufficient for the successful application of critical problem-solving skills when making clinical decisions [3]. Consequently, a variety of teaching modalities have been employed utilizing point-of-care ultrasound to develop clinical problem-solving skills. These tools include simulation, asynchronous teaching models, and hands-on scanning sessions. A review of recent literature has found that several programs have been successful in using ultrasound to teach medical students complex subject matters in various organ systems and clinical reasoning [4].

An area that has limited literature is Head, Eye, Ear, Nose, and Throat (HEENT) point-of-care ultrasound. Bernard et al. demonstrated that multimodal ultrasound instruction increased medical students' confidence in head and neck ultrasound [5]. We have developed a curriculum for HEENT ultrasound instruction that can be easily integrated into the medical school curriculum and correlates well with anatomy teaching. Our teaching allows the students to visualize and integrate basic anatomy to ultrasound findings and use the knowledge for clinical problem-solving. The objective of this study was to determine the feasibility of implementing point-of-care head and neck sonography and critical problem-solving instruction for medical student education.

## Materials and methods

Study design, setting, and participants

This cross-sectional study was conducted at an academic medical center. The study was approved by the institutional review board. Study participants were third-year medical students with minimal ultrasound experience. Participation in this study was voluntary, and students were informed that the decision to participate would not affect their academic grades. The medical students had only been previously exposed to a four-hour reproductive ultrasound skills workshop during their life cycle block, but otherwise had no integration of ultrasound into their curriculum in the first two years of medical school.

For our study, this was a one-day educational workshop focusing on the use of head and neck ultrasound for clinical problem-solving integrated into one of the week-long intersessions. The components of ultrasound training session included asynchronous learning, a didactic lecture, followed by a written pre-test assessment, a one-day hands-on workshop, and, finally, a follow-up assessment administered at the end of the workshop.

Study protocol

As part of the medical school curriculum for third-year medical students at our institution, students receive a one-week clinical rotation hiatus, called Intersessions, to learn multi-disciplinary topics to enrich their clinical experience. Our head and neck sonography curriculum was integrated into one of the week-long sessions, and it was conducted as a one-day educational training session focusing on the use of head and neck ultrasound for clinical problem-solving. The instructors for this workshop were composed of senior emergency medicine residents, emergency ultrasound fellows and faculty with emergency ultrasound fellowship training, or greater than 24 months of point-of-care ultrasound experience.

Head and neck sonography curriculum

1. Didactic session: Before the day of the hands-on ultrasound training session, the medical students were given a one-hour didactic lecture introducing the basics of HEENT ultrasound.

2. Asynchronous learning assignments: The medical students were also provided with a list of asynchronous learning assignments, pertaining to the various HEENT stations, and were asked to review them prior to the workshop. The learning assignments were short video podcasts reviewing the following topics: ultrasound-guided central line placement, peritonsillar abscess, ocular ultrasound, and sonographic confirmation of endotracheal tube placement.

**Figure 1:**
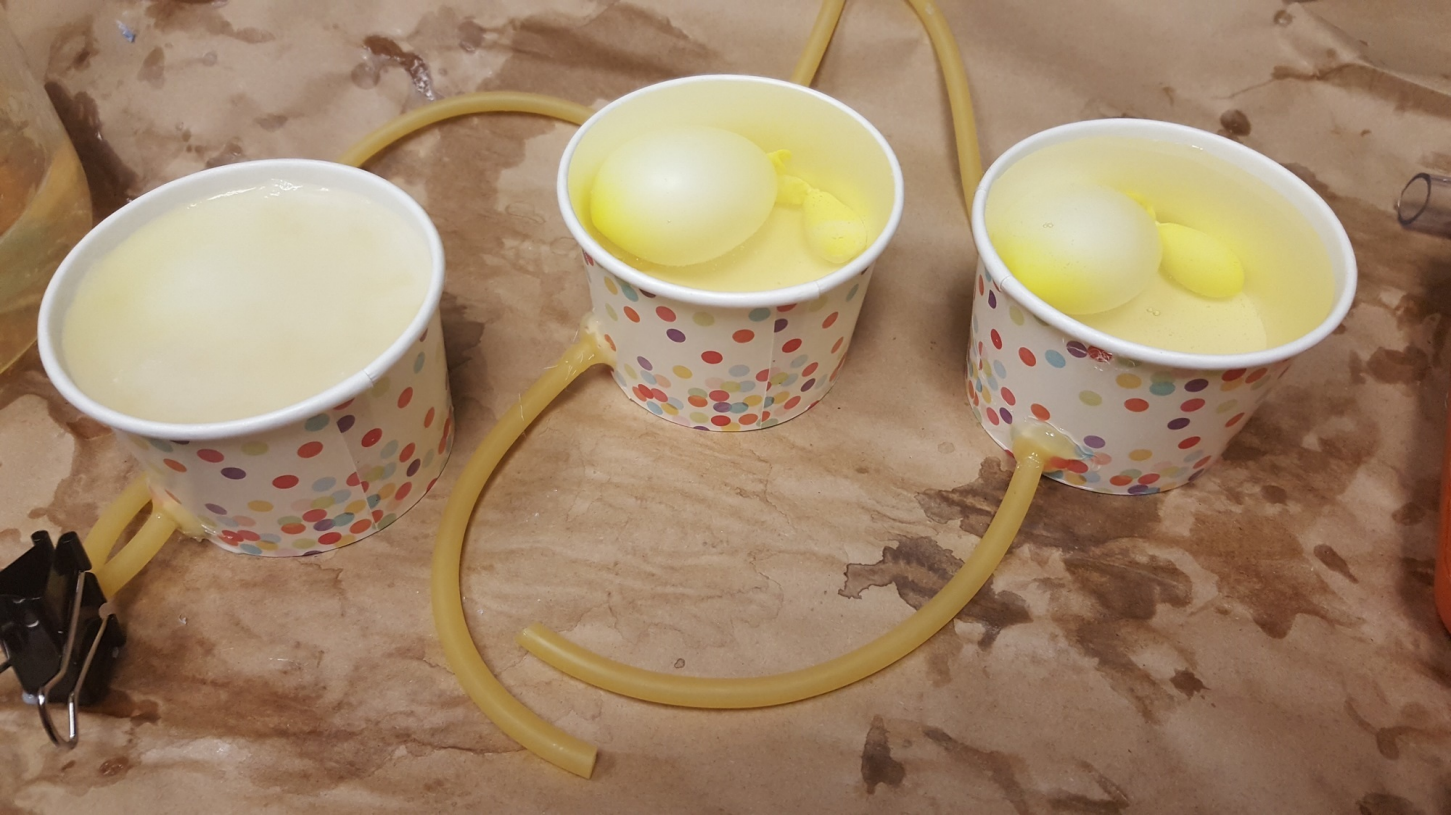
Peritonsillar abscess phantom

**Figure 2:**
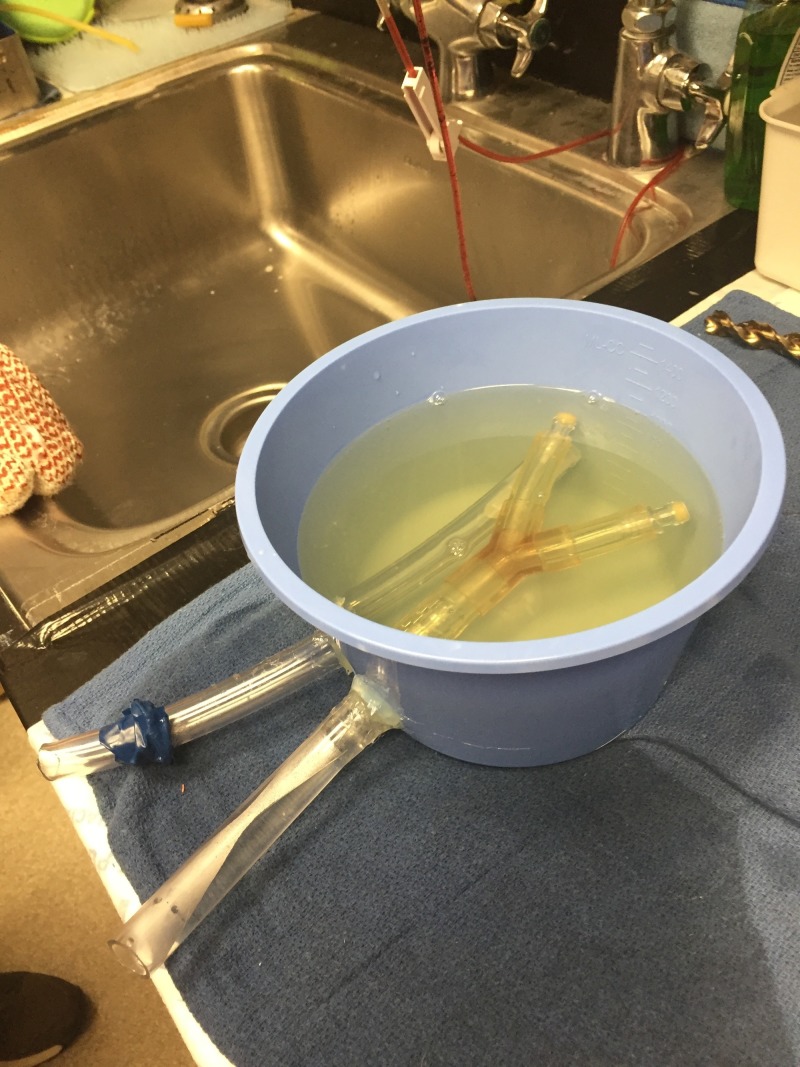
Airway phantom

3: Hands-on training: During the workshop, students were divided into groups of six to eight and rotated through hands-on educational ultrasound stations. Instruction provided during skill stations included different components of sonographic protocol, scanning technique, normal vs. abnormal findings, and clinical problem-solving using ultrasound skills and knowledge. An example of the clinical problem-solving that we used was if a patient had been intubated and became acutely hypoxic on the ventilator, students would use ultrasound to check for a pneumothorax and then check the placement of the endotracheal tube. The hands-on skill sessions were developed based on evidence-based HEENT applications and included: ocular ultrasound, sinusitis, dental abscess, peritonsillar abscess, endotracheal tube placement confirmation, thyroid/submandibular/parotid glandular appearance, and ultrasound-guided central line placement. Each station had unique learning objectives, and a description of the stations is summarized in Table 1. Each station was assigned an ultrasound machine and an instructor in no preferential order. In two stations, procedural skills were taught on phantoms developed at our institution (Figure 1 and Figure 2). A description of these models and a brief discussion of how to reproduce these phantoms is contained in Table 2.

**Table 1:** Skills station descriptions

Sonographic Skills Station	Description of Learning Objectives and Skills Station
Ocular Ultrasound: (30 minutes)	How to perform and what to look for during an ocular ultrasound This station included a review of normal ocular anatomy, and then students received a tutorial on how to perform an ocular ultrasound on a live model with normal anatomy. Sonographic video clips of ocular pathology (foreign bodies, globe rupture, afferent pupillary defect, lens dislocation, retinal detachment, vitreous hemorrhage, and increased intracranial pressure) were reviewed at the beginning of the session in PowerPoint format.
Upper Airway: (30 minutes)	How to perform and what to look for during upper airway ultrasound This station reviewed the basic anatomy of the upper airway, and then students received a tutorial on how to perform an upper airway ultrasound on a live model with normal anatomy. Sonographic video clips of upper airway pathology (sinusitis, dental abscess, peritonsillar abscess, and epiglottitis) were reviewed at the beginning of the session in PowerPoint format.
Head and Neck Glands: (30 minutes)	How to perform a head and neck gland ultrasound This station reviewed the basic anatomy of the head and neck glands, and then students received a tutorial on how to perform a head and neck gland ultrasound on a live model with normal anatomy. Sonographic video clips regarding the pathologic appearance of lymph nodes and salivary glands (parotid, submandibular, and thyroid) were reviewed in PowerPoint format.
Endotracheal Tube Confirmation: (30 minutes)	How to confirm the correct placement of an endotracheal tube during endotracheal intubation This station reviewed the sonographic anatomy of the esophagus and trachea as well as the skills necessary to confirm successful endotracheal intubation. Using a model created at our institution, the medical students were able to watch the endotracheal tube be placed under ultrasound to determine tracheal versus esophageal intubation. Students were also able to visualize the endotracheal cuff filled with saline to identify the endotracheal tube position within the trachea.
Sonographic Needle Guidance: (30 minutes)	How to guide needle placement for intravenous lines, central venous lines, etc. Intravenous phantom models were created for the medical students to learn ultrasound-guided needle procedures, akin to placing a central line or peripheral intravenous line under ultrasound guidance. They were also taught the sonographic appearance of the vein, artery, and nerve, along with the use of Doppler modes to identify the vessels on human models.

**Table 2:** Description of the phantoms used at each station

Sonographic Skills Station	Description of the Phantom Used
Peritonsillar Abscess Phantom	A peritonsillar abscess simulation model was created using small balloons filled with a thick echogenic liquid overlaid with ballistic gel and latex tubing connected to an intravenous fluid bag that could be compressed to simulate a pulsating internal carotid artery. The simulation model was then placed inside a mannequin head so the medical student can realistically learn how to insert and use the endocavitary probe to evaluate the oropharynx and identify a simulated peritonsillar abscess.
Endotracheal Tube Ultrasound Phantom	An airway simulation model was created for this station using a ridged plastic tube to simulate the tracheal structure with the cartilaginous rings. The ridged tube was divided into the right and left “main stem bronchus,” and a smooth plastic tube was placed slightly posterior and in the left-lateral position to the trachea model to simulate the “esophagus.” The medical students were able to watch the endotracheal tube be placed under ultrasound to determine trachea vs. esophagus and then visualize the endotracheal cuff filled with saline to identify the endotracheal tube position within the trachea.
Needle Guidance (Ultrasound for central lines and peripheral intravenous lines) phantom	Intravenous phantom models were created using ballistic gelatin and silicon tubing for the medical students to learn ultrasound-guided needle procedures, akin to placing a central line or peripheral intravenous line under ultrasound guidance.

Assessment

Medical students were asked to take a pre-test regarding the anatomy and pathology associated with the eye, esophagus, trachea, glands of the head and neck, tonsils, lymph nodes, and vasculature. The pre-test contained items focused on clinical problem-solving. Students also responded to questions regarding personal confidence in their sonographic skills of the aforementioned regions using a five-point Likert scale. After completing the training session, medical students were asked to complete the same questionnaires. The students’ performance and perceptions were compared before and after the training session.

Data analysis

All analyses were conducted in Stata 11 (StataCorp LP, College Station, TX, US). Data are presented as means with standard deviations (SD) and percentages with 95% confidence intervals (CI).

## Results

 A total of 123 subjects participated in this head and neck sonography training session. Ninety-one percent completed the pretest questionnaire prior to the workshop, and 83% completed the post-test questionnaire. The students’ comfort level with using an ultrasound system significantly increased from 31% to 92%. Additionally, the comfort level in interpreting ultrasound images also significantly increased from 21% to 84%. Eighty-nine percent (95% CI, 86%-97%) had an interest in learning ultrasound and would enroll in an optional ultrasound curriculum if given the opportunity. Knowledge of specific ultrasound applications also increased from 60% (after asynchronous learning and the lecture) to 95% (after additional hands-on sonographic training). Table 3 summarizes performance in the pretest and post-test questionnaires testing the knowledge of specific ultrasound applications.

**Table 3:** Differences in performance in pretest and posttest questionnaires testing knowledge related to specific ultrasound applications.

Specific Ultrasound Applications	Pre-test (n=112) Students Accurately Answered the Test Items (%)	Post-test (n=103) Students Accurately Answered the Test Items (%)	Differences in Performance (%)	p-value
Ocular				
Sonographic anatomy	61.6	95.2	33.6	<0.001
Sonographic characteristics of the eye	97.3	100	2.7	0.69
Retinal detachment identification	58.0	81.6	23.6	<0.001
Signs of elevated intracranial pressure	69.6	89.3	19.7	0.004
Airway				
Sonographic appearance of trachea Sonographic appearance of pleura	42.3 77.3	39.2 90.3	-3.1 13	0.65 0.06
Sonographic anatomy of the lung	91.9	99.0	7.1	0.30
Head, Eye, Ear, Nose and Throat Infections				
Sonographic anatomy of the neck	92.8	94.1	1.3	0.85
Diagnostic accuracy of clinical assessment for a peritonsillar abscess	55.0	80.6	25.6	<0.001
Sonographic appearance of the abscess	52.3	75.7	23.4	<0.001
Sonographic appearance of parotid cellulitis	70.9	80.2	9.3	0.18
Head, Eye, Ear, Nose and Throat Glands				
Sonographic anatomy	81.1	96.1	15	0.03
Artifacts	84.7	81.6	-3.1	0.65
Sonographic characteristics of lymphadenitis	47.8	58.3	10.5	0.13
Sonographic characteristics of sialolithiasis	63.6	71.8	8.2	0.23
Head, Eye, Ear, Nose, and Throat Vascular Access				
Sonographic characteristics of blood vessels	86.6	90.3	3.7	0.59
Sonographic anatomy of veins	97.3	98.1	0.8	0.91
Uses of color Doppler	79.5	86.4	6.9	0.31
Needle insertion approach	75.0	94.2	19.2	0.01
Needle visualization under ultrasound	41.4	35.9	-5.5	0.42

## Discussion

Medical students perceive ultrasound training as a valuable tool in understanding human anatomy, learning physical examination skills, and applying the skillset to clinical reasoning and performing invasive procedures. Prior studies have demonstrated that medical students are able to learn basic ultrasound techniques after focused training sessions [6]. With the widespread use of bedside ultrasound in a wide range of medical specialties, medical students would benefit from early exposure and training in ultrasound. Depending on the availability of resources, a variety of techniques are currently used to teach ultrasound to medical students. Although revamping the current anatomy education may be difficult, the integration of an educational intervention similar to that of our study can be a practical way to increase applied anatomy education during the clinical components of undergraduate medical education. Theme-based or anatomical region-based interventions, such as the one detailed in this study, may provide the exposure and education necessary for clinical problem-solving and integrated applied anatomy education. Ultrasound is the ideal imaging modality to help students concurrently learn clinical pathology and clinically relevant anatomy. Focused training sessions highlighting the use of point-of-care ultrasound as a clinical problem-solving tool can be a useful way to integrate this educational modality. In this study, we focused on the sonography of the head and neck and integrated this educational intervention during the clinical third year of medical training. We specifically focused on the clinical evaluation of HEENT emergencies and our curriculum was well-received by medical students. The emphasis of our training session was on the integration of ultrasound concepts with other elements of clinical assessment. Similar techniques can be utilized to teach medical students other ultrasound applications.

In our study, a variety of HEENT applications were covered in the curriculum (ocular, airway, HEENT infections/glands, and HEENT vascular access) and knowledge before and after the training session was compared. Students demonstrated an improvement in test scores related to HEENT infections and ocular applications. A statistically significant improvement in scores was not consistently seen with the HEENT glands, airway, and vascular applications. These findings will guide us in revising the future curriculum. For example, students did not significantly improve their scores when testing the airway application. This suggests that we need to revisit how the information is presented in the didactic session and asynchronous learning assignments. These results also provide us with the opportunity to improve our phantoms. For example, this was the first opportunity we had to use the airway phantom that was created at our institution. Our results can reveal aspects of this new phantom that are amenable to improvement. The insignificant improvement in testing in regards to HEENT vascular access can be explained by the students’ lack of procedural skills and ultrasound device familiarity as third-year medical students. Similar to prior studies, our students also expressed an interest in learning ultrasound and would enroll in an optional ultrasound curriculum if given the opportunity. The increase in students’ overall comfort level with using an ultrasound system and interpreting ultrasound images suggest that focused training sessions are effective in teaching advanced ultrasound applications.

This study has several limitations. First, our data represents students at a single institution, so the generalizability of the study may be limited. This is also an institution with an emergency ultrasound fellowship program with resources that other medical schools may not have such as instructors and expertise. The questionnaires were not piloted before the session, and there was no comparator. The station instructors had various levels of ultrasound experience, spanning from senior emergency medicine residents to more experienced emergency ultrasound faculty members. There were fewer participants in the post-test questionnaire than the pre-test, so the direct comparison of the pre-test and post-test scores may be flawed. Multiple phantom models were created for this teaching day, and because it is impossible to subject a live model to invasive procedures and ultrasound evaluations, the medical students learned endotracheal tube placement, peritonsillar abscess evaluation, and needle guidance on phantoms that did not exactly reproduce the feel and texture of a live model.

## Conclusions

At our institution, we successfully integrated point-of-care head and neck sonography and critical problem-solving instruction for medical student education. The students’ comfort level with using an ultrasound system and interpreting ultrasound images significantly increased after the training session. Our head and neck sonography curriculum also resulted in an improvement in the knowledge of head and neck ultrasound applications.
